# EXPLORING THE IMPACT OF AN EBOOK CLUB PROGRAM ON APATHY AMONG OLDER CANADIANS IN LONG-TERM CARE: A FEASIBILITY STUDY

**DOI:** 10.1093/geroni/igad104.2289

**Published:** 2023-12-21

**Authors:** Aderonke Agboji, Shannon Freeman

**Affiliations:** University of Northern British Columbia, Prince George, British Columbia, Canada; University of Northern British Columbia, Prince George, British Columbia, Canada

## Abstract

Apathy is a common and persistent problem among older people in institutional settings. Research has shown that apathy can lead to rapid decline in cognitive status, serious functional impairments and decrease in the life span of those affected, yet it is under-researched, and under-managed. The aim of this study was to explore ways by which apathy can be mitigated in this population using eReader technology during book club program implementation. We recruited participants from various long-term care facilities (N=18) and each participants took part in semi-structured interviews, and self-reported apathy assessment both at the start and end of the program. We tracked engagement in the eBook club and time commitment to reading using a logbook. The findings suggest that older people, including persons with mild to moderate dementia, are open to adopting new technologies and the use of eReaders during book club programs is feasible for long term care facilities. Overall, we found that the use of eReader technology is a safer and more effective way of delivering book club program to older people in long term care facilities particularly during pandemic such as COVID 19 as they are easy to disinfect in comparison to physical books. We recommend that long term care staff should adapt eReaders into their existing book club or literacy programs and consider utilizing it as a strategy to prevent or manage apathy in this population.

